# Fas Ligand enhances vessel maturation and inhibits vascular leakage associated with age-related macular degeneration

**DOI:** 10.3389/fimmu.2026.1898554

**Published:** 2026-10-22

**Authors:** Adarsha Koirala, Ann Marshak-Rothstein, Bruce R. Ksander, Meredith Gregory-Ksander

**Affiliations:** 1 Schepens Eye Research Institute of Massachusetts Eye and Ear Infirmary, Department of Ophthalmology, Harvard Medical School, Boston, MA, United States; 2 Department of Medicine, University of Massachusetts (UMass) Chan Medical School, Worcester, MA, United States

**Keywords:** age-related macular degeneration, choroidal neovascularization, Fas Ligand, macrophage, myeloid cells, vascular leakage, vessel normalization

## Abstract

**Introduction:**

Age-related macular degeneration (AMD), results from choroidal neovascularization (CNV), retinal edema and loss of photoreceptors. Previous studies suggest that Fas Ligand (FasL) on retinal pigment epithelial cells inhibits CNV by inducing apoptosis of infiltrating Fas+ vascular endothelial cells. However, membrane-bound FasL (mFasL) can be cleaved by metalloproteinases to release a soluble fragment (sFasL) and the two isoforms have distinct functions. mFasL induces apoptosis and inflammation while sFasL has been shown to promote cell activation, cell survival, cell proliferation, and cell migration. In this current study, we use a genetic approach to prevent cleavage of mFasL in order to determine the extent to which sFasL and/or mFasL contribute to vascular leakage in a mouse model of laser-induced CNV.

**Methods:**

To distinguish the role of mFasL from sFasL in regulating CNV and vascular leakage, we used a 532 nm laser to induce CNV in three types of mice: (i) wild-type (WT) FasL^WT/WT^ mice that express low levels of mFasL and high levels of sFasL, (ii) homozygous FasL^ΔCS/ΔCS^ mice that express greater than normal levels of mFasL and no sFasL, and (iii) heterozygous FasL^ΔCS/WT^ mice that express greater than normal levels of mFasL and less than normal levels of sFasL.

**Results:**

Unexpectedly, CNV size and the area of vascular leakage were comparable in homozygous FasL^ΔCS/ΔCS^ and WT mice. However, heterozygous FasL^ΔCS/WT^ mice developed significantly less vascular leakage and showed accelerated neovessel maturation that depended on the expression of the Fas receptor in LysM-positive myeloid cells. Moreover, the protective phenotype observed in heterozygous FasL^ΔCS/WT^ mice correlated with increased retinal expression of PDGFRb and TGFβ1, reduced retinal expression of VEGFA, and increased expression of PEDF, which is known to inhibit VEGF-induced vascular permeability and promote vessel normalization.

**Discussion:**

Overall, these data indicate that FasL-mediated protection from CNV depends on expression of both sFasL and mFasL. Moreover, the accelerated neovessel maturation observed in heterozygous FasL^ΔCS/WT^ mice depends on Fas signaling in LysM-positive myeloid cells and is highly dependent on the extent of FasL cleavage, identifying Fas ligand cleavage as a potential therapeutic strategy to promote neovessel maturation and prevent vascular leakage in wet AMD.

## 1 Introduction

Age-related macular degeneration (AMD) is an irreversible blinding disease that manifests as loss of central vision primarily in the older population (> 50 years of age) (1). Approximately 200 million people worldwide currently suffer from AMD, a number that is expected to increase to 288 million by 2040 (2). AMD is associated with the breakdown of the outer blood-retina-barrier and subsequent choroidal neovascularization (CNV) within the macula. The subsequent formation of immature choroidal neovessels leads to vascular leakage, retinal edema and the death of photoreceptors. The current anti-vascular endothelial growth factor (VEGF) antibody therapy for neovascular AMD has been quite successful at preventing vascular leakage, resolving retinal edema, and preventing vision loss (3, 4). However, this treatment requires long-term inhibition of VEGF by repeated intravitreal injections of an anti-VEGF antibody, and if treatment is discontinued disease progression resumes. Since VEGF is also required to maintain normal retinal homeostasis and is secreted by many cell types within the retina (5–7), the long-term inhibition of VEGF is predicted to have significant negative consequences such as impaired ciliary body and Müller cell function (7, 8). In addition, a significant number of wet AMD patients do not respond to anti-VEGF treatment while other patients who initially respond to treatment gradually become unresponsive after an extended treatment regime (9, 10). Treatment associated complications such as hemorrhagic lesions (11) and outer retinal atrophy (3, 9, 12–14) have also been reported. Finally, the repeated intravitreal injections required for effective treatment place a substantial burden on patients, often leading to poor adherence and progressive vision loss. For these reasons, there is a need for improved alternative approaches to treat neovascular AMD.

An alternative strategy could take advantage of the regulatory capacity of ocular Fas ligand (FasL). FasL is a transmembrane protein and a member of the TNF family that can induce both apoptosis and the production of proinflammatory cytokines (15). FasL was first implicated in ocular immune privilege by the now classical ACAID studies of Dr. Wayne Streilein and colleagues (16–19). A considerable research effort has since focused on the function of FasL in the development and regulation of angiogenesis in the retina. The importance of FasL in controlling the growth and development of CNV was first illustrated in FasL-defective (gld) mice that developed more severe laser-induced CNV than wild-type mice (20). It was proposed that membrane-bound FasL (mFasL) expressed by RPE cells triggered apoptosis of infiltrating Fas+ vascular endothelial cells, thus inhibiting angiogenesis and the subsequent development of CNV (21–23).

However, mFasL can be cleaved by metalloproteinases to release a soluble fragment (sFasL) that can trimerize to engage Fas trimers but fails to induce cell death or the production of proinflammatory cytokines. FasL-induced apoptosis requires extensive multimerization and binding to multimerized Fas that accumulates in lipid rafts (24, 25). In contrast to mFasL, sFasL has been reported to promote cell activation, cell survival, cell proliferation, and cell migration and (26–32) The relevance of FasL cleavage in AMD-associated CNV was previously investigated using systemic administration of doxycycline, a metalloproteinase (MMP) inhibitor, to block the cleavage of mFasL (33). Doxycycline treated mice were shown to develop smaller CNV lesions in a laser-induced model of AMD. Reduced angiogenesis correlated with increased FasL expression on choroidal tissue and decreased sFasL in the serum and eye as measured by ELISA, consistent with the notion that decreased cleavage led to increased expression of pro-apoptotic mFasL. Nevertheless, while doxycycline can block proteolytic FasL cleavage, it also has a variety of other anti-inflammatory and anti-angiogenic effects that are independent of the Fas/FasL pathway (34). Moreover, doxycycline may not block all FasL-cleaving enzymes. In fact, examination of surgically excised choroidal neovascular membranes from patients diagnosed with wet AMD did not find a correlation between the expression of mFasL on RPE cells and either the size of neovascular membranes or the extent of choroidal endothelial cell apoptosis (35, 36).

To avoid confounding problems associated with MMP inhibitors, we decided to use a genetic approach that would allow us to more specifically control the extent of mFasL cleavage. C57BL/6 mice with a gene-targeted mutation of the FasL metalloproteinase cleavage site (ΔCS mice) have previously been used to examine the role of mFasL and sFasL in murine glaucoma (30). This mutation does not affect transcription or translation of FasL, or cell types that do not express FasL. The difference between wild-type (FasL^WT/WT^) and FasL^ΔCS/ΔCS^ mice is simply that activated FasL^ΔCS/ΔCS^ cells express higher levels of mFasL and no sFasL (30). Therefore, if FasL simply prevents the outgrowth of new endothelial cells, then FasL^ΔCS/ΔCS^ mice should be better protected from CNV than WT mice. Moreover, FasL^ΔCS/WT^ heterozygous mice should have an intermediate phenotype.

Intriguingly, we now show that expression of mFasL alone does not inhibit the development of CNV in a mouse model of laser induced CNV, which is not consistent with a purely mFasL-mediated endothelial apoptosis model. Rather, our data suggest that FasL may prevent vascular leakage through a *non*apoptotic pathway that promotes accelerated neovessel maturation. Furthermore, we demonstrate that accelerated neovessel maturation depends on Fas signaling in myeloid cells and is highly dependent on the extent of FasL cleavage. Importantly, these studies identify modulation of Fas ligand cleavage as a potential new therapeutic strategy to promote neovessel maturation and prevent vascular leakage in wet AMD.

## 2 Materials and methods

### 2.1 Animals

All animal experiments were approved by the Institutional Animal Care and Use Committee at Schepens Eye Research Institute, Boston, MA, and were performed under the guidelines of the Association for Research in Vision and Ophthalmology Resolution on the Use of Animals in Research. All mice were housed in an AAALAC approved animal facility at Schepens Eye Research Institute, given standard mouse chow and water ad libitum, and maintained on a 12 hr light/dark cycle. Both male and female littermates (FasL ^WT/WT^, FasL^ΔCS/ΔCS^, FasL^ΔCS/WT^) at 10–12 weeks old were used in this study. However, while both genders were equally represented across experimental groups (**Supplementary Table 1**), the study was not designed or sufficiently powered to assess sex-specific effects. The FasL^ΔCS/ΔCS^ mice were generated as described previously (30). The founder mice were backcrossed for 10 generations onto the C57BL/6J background to generate the FasL^WT/WT^, FasL^ΔCS/ΔCS^, and FasL^ΔCS/WT^ mice used in the studies described herein. To generate heterozygous FasL^ΔCS/WT^ mice that conditionally lack Fas only in the myeloid cell compartment, FasL^ΔCS/ΔCS^ mice were intercrossed with C57BL/6 mice homozygous for the loxP flanked Fas allele (Fas^flox/flox^, Jackson Laboratory #007895) to generate mice that were homozygous for the non-cleavable ΔCS FasL allele and homozygous for the loxP flanked Fas allele and (FasL^ΔCS/ΔCS^Fas^flox/flox^). The FasL^ΔCS/ΔCS^Fas^floox/flox^ mice were then further crossed to mice expressing Cre under control of murine lysozyme M gene promoter (Cre^Lysm^; The Jackson Laboratory), thereby generating FasL^ΔCS/WT^Cre^Lysm^ Fas^flox/flox^ mice. All mice were anesthetized (for laser photocoagulation, SD-OCT, fluorescein angiography, and vascular perfusion) by intraperitoneal (IP) injection of ketamine (100mg/kg body weight) and Xylazine (20mg/kg body weight). Mice were euthanized (for tissue harvest) by CO_2_ inhalation (flow rate = 1.8L/min, 30% volume displacement per minute) followed by cervical dislocation.

### 2.2 Laser photocoagulation

Laser photocoagulation was performed in one eye per mouse, using a 532-nm laser (Meridian) attached to the Micron III fundus camera (Phoenix Research Labs, Pleasanton, CA, USA). Following anesthesia, the pupils were dilated with topical application of 1% tropicamide. The fundus imager was used to focus the laser beam on the junction of the RPE/choroid. Four lesions were created using a pulse duration of 50 ms, -100 mW, and 50 um spot size at the 3, 6, 9, and 12’0 clock meridians centered on the optic nerve and located two-or three disc diameters from the optic nerve.

### 2.3 Spectral Domain Optical coherence tomography

The size of CNV lesions was determined using a SD-OCT system (Bioptigen, Durham, NC). Pupils of anesthetized mice were dilated with topical application of 1% tropicamide. Genteal (Novartis) was applied to hydrate the ocular surface and maintain corneal clarity. Volume analysis centered on the optic nerve head was performed, using 100 horizontal, raster, and consecutive B-scan lines, each one composed by 1200 A- scans. *In Vivo* Vue 2.0 software was used to acquire images. Quantification of lesion size by OCT was performed using ImageJ software from NIH as described previously (37). Briefly, OCT images were taken through the center of each lesion, defined as the midline passing through the area of the RPE-Bruch’s membrane rupture. Individuals performing the OCT imaging were blinded to the experimental groups. The lesion size was calculated by outlining the entire area occupied by lesion and leakage using ImageJ software.

### 2.4 Fundus imaging and fluorescein angiography

Fundus imaging was performed using the MICRON III camera (Phoenix Laboratories, CA, USA). Pupils of anesthetized mice were dilated with topical application of 1% tropicamide. Genteal (Novartis) was applied to maintain hydration of the ocular surface. Images were acquired using brightfield or GFP filters (excitation, 482 nm, emission, 536 nm). FA was performed following ip injection of 1% fluorescein (HUB pharmaceuticals, LLC, Rancho Cucamonga, CA) at a dose of 0.2ml/25 g body weight and the individuals performing the FA were blinded to the experimental groups. FA images were taken at three different time points: 1–2 mins, 4–5 mins and 9–10 mins post ip-injection of fluorescein. The final images taken at 9–10 mins were turned into black and white images; and using contrast, the total white area was measured using ImageJ software and represented the total area of vascular leakage. Lesions were also graded using a previously established grading system (38). Grade 0 (not leaky) faint hyperfluorescence or mottled fluorescence, no leakage; Grade 1 (questionable leakage) hyperfluorescent lesion without progressive increase in size or intensity; Grade 2A (leaky) hyperfluorescence increasing in intensity but not in size; Grade 2B (Clinically significant leakage) hyperfluorescence increasing in intensity and size.

### 2.5 FITC-dextran perfusion

At the time point for euthanasia anesthetized mice were perfused through the left ventricle with 10 ml of 1xPBS followed by 7 ml 5 mg/ml FITC-dextran (Mol. Wt. 2 x 10^6^; Sigma, St. Louis, MO). Following perfusion, the eyes were enucleated and processed for immunohistochemistry.

### 2.6 Immunohistochemistry

Posterior eyecups (retina, RPE, choroid and sclera) were fixed in 4% paraformaldehyde (PFA) for 2 hours and embedded in OCT media for frozen sections or placed in 1xPBS overnight for choroidal whole-mount preparations. Retinal frozen sections or choroidal whole mounts were then incubated in blocking solution (0.5% BSA, 0.2% Triton in 1xPBS) for 1 hour at RT and incubated overnight with the primary antibody. Collagen IV (Abcam-ab19808) was used at 1:100, NG2 (Abcam-ab83178) at 1:100, cd31 (BD Pharmingen cat #550274) at 1:50 concentrations. AlexaFluor secondary antibodies were used at 1:500. Confocal images were acquired with a Leica confocal microscope and processed using the SP6 software. Collagen IV staining was used to assess total lesion size and FITC-dextran perfusion was used to assess total area of perfused vessels. The % area of fluorescence per lesion was measured using ImageJ software.

### 2.7 Electron microscopy

Eyes were enucleated at 5 days post laser and cornea, iris and lens were removed and posterior eyecups (retina, RPE, choroid and sclera) were immersed in the Karnovsky’s fixative (2% formaldehyde + 2.5% glutaraldehyde, in 0.1 M sodium cacodylate buffer, pH 7.4 (Electron Microscopy Sciences, Hatfield, Pennsylvania) for a minimum of 12 hours at 4 °C. Following fixation, samples were rinsed with 0.1M sodium cacodylate buffer, post-fixed with 2% osmium tetroxide in 0.1M sodium cacodylate buffer, en bloc stained with 2% aqueous uranyl acetate, then dehydrated with graded ethyl alcohol solutions, transitioned with propylene oxide and resin infiltrated in tEPON-812 epoxy resin (Tousimis, Rockville, Maryland) utilizing an automated EMS Lynx 1 EM tissue processor (Electron Microscopy Sciences, Hatfield, Pennsylvania). Processed tissues were oriented in tEPON-812 epoxy resin and polymerized in silicone molds using an oven set for 60 °C. Semi-thin cross-sections for light microscopy were cut at 1-micron and stained with 1% toluidine blue in 1% sodium tetraborate aqueous solution for assessment and screening regions of the tissue block face for thin sectioning. Ultrathin sections (60–90 nm) were cut from the epoxy block using a Leica EM UC7 ultramicrotome (Leica Microsystems, Buffalo Grove, IL) and a diamond knife, collected onto grids that were stained with aqueous 2% uranyl acetate and Sato’s Lead citrate. Grids were imaged using a FEI Tecnai G2 Spirit transmission electron microscope (FEI, Hillsboro, Oregon) at 8o kV interfaced with an AMT XR41 digital CCD camera (Advanced Microscopy Techniques, Woburn, Massachusetts) for digital TIFF file image acquisition.

### 2.8 Western blot and ELISA

Eyes were enucleated and placed in ice-cold 1 x PBS with protease inhibitor cocktail (pH 7.5; Roche Chemicals, Basel, Switzerland). The anterior segment, including the cornea, iris, ciliary body, and lens were removed. The posterior eyecups (2 eye cups per sample) consisting of the retina, choroid, and RPE were homogenized using a pestle pellet homogenizer in 100ul RIPA buffer. The buffer was then collected, pooled and centrifuged at 13,000 rpm. Protein concentration was then determined using the Nanodrop 2000c (Thermo Scientific) and supernatants were used for Western Blot and ELISA. Invitrogen NuPage LDS sample buffer was used for loading in a 12% gel for Western Blot analysis. Transfer was performed using the iBlot dry transfer equipment from Invitrogen. PEDF antibody (Millipore cat # 07-280) was used at 1:100 concentrations, actin antibody 1:50 (sc1615, Santa Cruz). LI-COR secondary antibodies were used for detection according to manufacturer’s protocol. Odyssey CLx Infrared imaging system (LI- COR Biosciences, USA) was used to visualize the bands. Mouse VEGF ELISA kit from Sigma (cat# RAB0509-1KT) was used to quantify VEGF levels according to the manufacturer’s protocol.

### 2.9 Quantitative real time PCR

Eyes were enucleated and RNA was isolated from posterior eyecups (retina, choroid, and sclera) using Qiagen RNeasy mini kit (Cat # 74104) according to the manufacturer’s protocol. RNA was treated with DNAse (Cat # AM222, Invitrogen) to ensure no contamination of genomic DNA. 500ng of RNA was used to prepare cDNA using the iScript cDNA synthesis kit from Bio-Rad (Cat # 170-8890) according to the manufacturer’s protocol. cDNA was treated with RNaseH (18021-014, Invitrogen) to ensure absence of single stranded RNA. Quantitative real time PCR was performed using the master mix from Invitrogen (Cat # 4472903) using the manufacturer’s protocol in 10μl total volume in duplicates. Relative expression to housekeeping gene β-actin was quantified using the formula: Relative expression =10,000 x 1/2^(Avg. Gene cT- Avg. β-actin cT). Fold changes were calculated with respect to untreated control eyes. The primers used include: β- actin- F-5’ AAATCGTGCGTGACATCAAA-3’R-5’ AAGGAAGGCTGGAAAAGAGC 3’, TGFβ1-F- 5’ GCTGCGCTTGCAGAGATTAAA 3’R-5’-TTGCTGTACTGTGTGTCCAG 3’, PDGFb- F-5’-ACTCCATCCGCTCCTTTGAT- 3’R-5’-GTCTTGCACTCGGCGATTA- 3’ and PDGFbR- F-5’ CAACCGTACCTTGGGTGACT 3’R-5’-GAGAGCTGGACCTCATCGTC 3’.

### 2.10 Statistical analyses

Graph pad prism 6 (La Jolla, CA, USA) was used for statistics. Statistics were performed as indicated in each figure legend using two-way ANOVA and Bonferroni’s *post-hoc* comparisons or one-way ANOVA and Tukey’s *post-hoc* comparisons. Significance was defined at a P value <0.05.

## 3 Results

### 3.1 Membrane FasL alone does not inhibit laser-induced CNV

We have previously shown that FasL is constitutively cleaved in healthy eyes (39), so to determine how the failure to cleave FasL would impact the extent of CNV and vascular leakage in a model of AMD, we used a genetic approach that would allow us to specifically control the extent of mFasL cleavage and therefore modulate the levels of mFasL and sFasL. In this study, we used C57BL/6 mice with a gene-targeted mutation of the FasL metalloproteinase cleavage site (ΔCS mice) that have previously been used to examine the role of mFasL and sFasL in murine glaucoma (30). The homozygous FasL^ΔCS/ΔCS^ mutant mice express increased levels of mFasL and no detectable sFasL. In this study we used three groups of mice: (A) wild-type FasL^WT/WT^ mice where FasL is primarily expressed as the cleaved sFasL fragment (39), (B) homozygous FasL^ΔCS/ΔCS^ mice (both FasL alleles possess the ΔCS mutation) that will express only mFasL and no sFasL (28, 30), and (C) heterozygous FasL^ΔCS/WT^ mice (one FasL allele possesses the ΔCS mutation and one FasL allele is WT) that will express greater than WT levels of mFasL and less than WT levels of sFasL (**Figure 1**).

**Figure 1:**
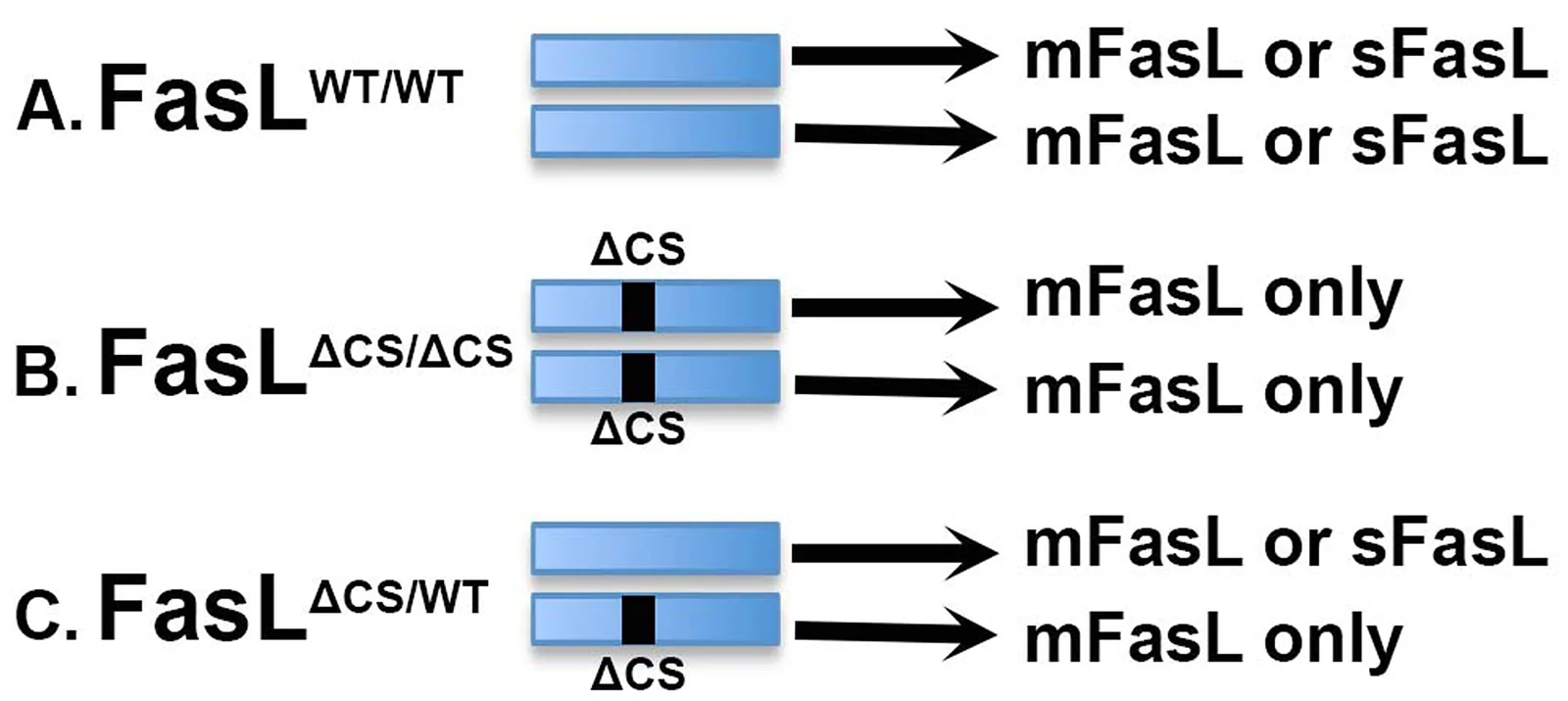
FasL alleles diagram. **(A)** Wild-type FasL^WT/WT^ mice in which both FasL alleles are wild-type and express low levels mFasL and high sFasL, **(B)** homozygous FasL^ΔCS/ΔCS^ mice in which both FasL alleles possess the ΔCS mutation (black line) and express greater than normal levels of mFasL and no sFasL and **(C)** heterozygous FasL^ΔCS/WT^ mice in which only one FasL allele possesses the ΔCS mutation and express greater than normal levels of mFasL and less than normal levels of sFasL as compared to WT mice (> normal mFasL and < normal sFasL).

Laser photocoagulation of the retina produces an immediate injury (Day 0) (**Figures 2A–C**) that can be visualized by SD-OCT (**Figure 2D**) and fluoresceine angiography (FA) (**Figures 2E, F**), followed by the development of CNV between days 3–14 post laser treatment (37, 40). Using an image-guided laser system, we were able to precisely and reproducibly control location, size, and intensity of the laser photocoagulation required for CNV. We first confirmed the initial laser-induced injury was uniform across all three cohorts (FasL^WT/WT^ FasL^ΔCS/^WT, and FasL^ΔCS/^ΔCS) using SD-OCT (**Figures 2G, H**) and FA (**Figures 2I, J**). As previously reported (37, 40), in the laser-induced mouse model of CNV, rupture of Bruch’s membrane produces a cavitation bubble that appears on SD-OCT as a subretinal hyporeflective lesion on Day 0 that progresses to a larger, butterfly-like lesion by Day 2 (**Figure 2G**). By contrast, fluorescein angiography (FA) performed immediately after laser injury (Day 0) reveals a bright area of hyperfluorescence resulting from the acute rupture of Bruch’s membrane and leakage of fluorescein from the damaged choroidal vasculature (**Figure 2I**). Quantification of the lesion size by SD-OCT (**Figure 2H**) and area of leakage by FA (**Figure 2J**) demonstrate the initial laser-induced injury was uniform across all three cohorts (FasL^WT/^WT, FasL^ΔCS/WT,^and FasL^ΔCS/ΔCS^ mice). The reduction in fluorescence detected on Day 2 post laser injury (**Figure 2I**), represents the clotting and closure of the acutely damaged choroidal vessels. Taken together, we conclude that the partial (FasL^ΔCS/WT^) or complete (FasL^ΔCS/ΔCS^) prevention of FasL cleavage did not affect the initial laser-induced injury, resulting in uniform retinal lesions across all three cohorts.

**Figure 2:**
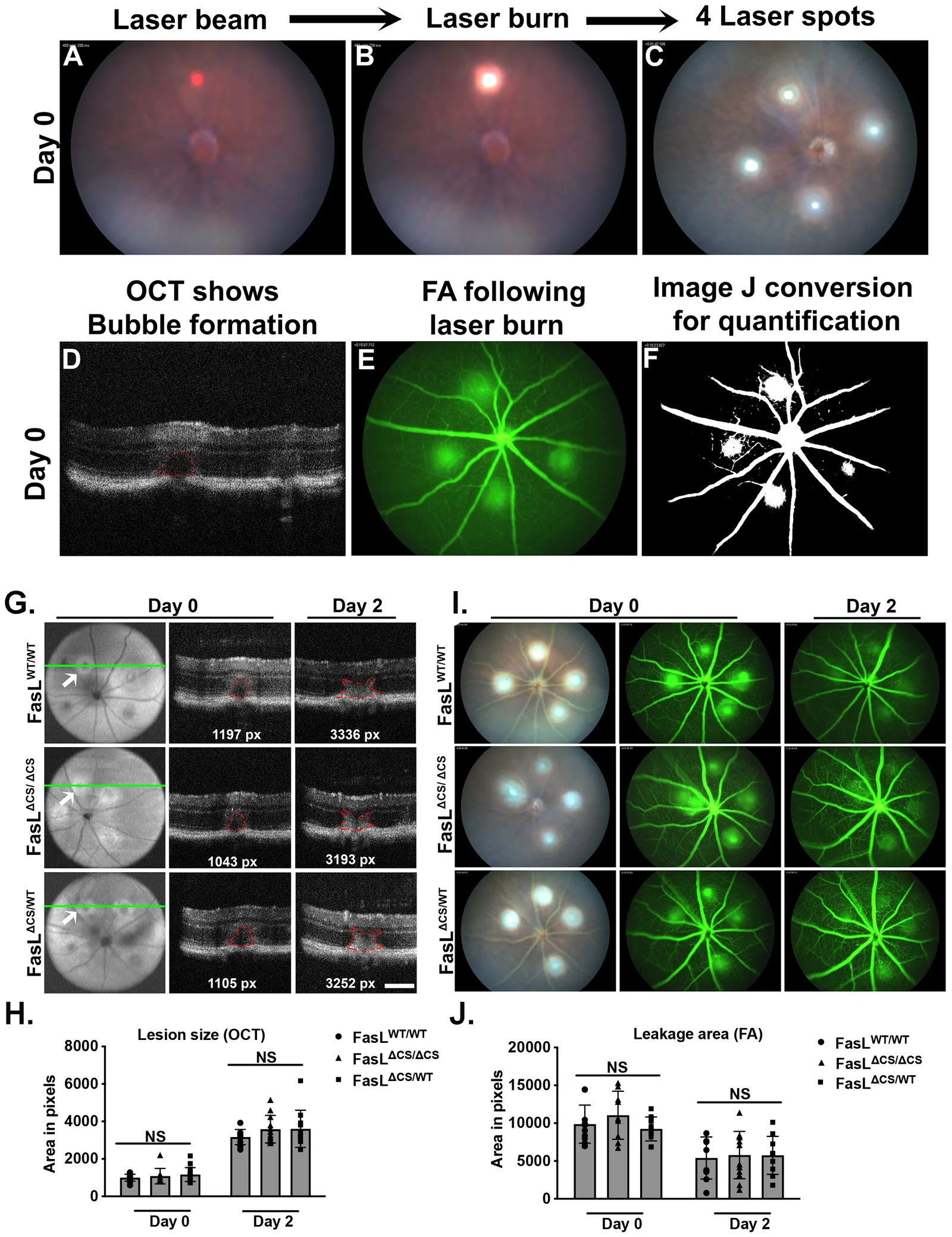
Evaluating consistency of laser-photocoagulation by SD-OCT and Fluorescein Angiography. **(A)** Laser injector was attached to Micron III fundus camera to place a laser beam directed to the RPE/Bruch’s junction to form laser spots, followed by **(B)** Laser burns. **(C)** Fundus image depicting all 4 laser spots following laser burns. **(D)** OCT b-scan acquired immediately post laser burn. **(E)** FA image acquired 4–5 minutes post i.p. injection of Fluorescein immediately post laser burn. **(F)** Image processing by Image J for quantification of size of the initial photocoagulation. **(G)** OCT images acquired at day 0 and 2 post laser. Left panel, a volume scan at day 0 post laser identifying the center of the lesion (green line) where the central hyporeflective area confirms a break in Bruch’s membrane. Middle panel, a rectangular b-scan taken through the center of the lesion (lesion identified by arrow) at day 0 shows the formation of the subretinal bubble immediately after laser injury (outlined in red, area in pixels). Right panel, a rectangular b-scan at day 2 shows the development of the characteristic butterfly-like lesion (outlined in red, area in pixels) (Scale bar = 0.1mm). Representative OCT images at Day 0 and Day 2 post laser from one animal in each group. **(H)** Quantification of subretinal bubble and butterfly-like lesion was performed using OCT scans at day 0 and 2 post laser. Shown is the mean area in pixels ± SD, 10–14 mice per group from 3 independent experiments **(I)** Fluorescein angiography performed at day 0 and 2 post laser. Left panel (day 0, bright field image), Middle panel (day 0, green filter). Right panel, (day-2, green filter). Representative fluorescein angiography images at Day 0 and Day 2 post laser from one animal in each group. **(J)** Quantification of the area of photocoagulation at day 0 and day 2 post laser. Shown is the mean area in pixels ± SD, 10–16 mice per group from 3 independent experiments. Statistical analysis was performed using 2-way ANOVA and Bonferroni’s multiple comparisons tests.

To examine CNV development in these three cohorts of mice, we then determined the size of CNV lesion by SD-OCT and the area of vascular leakage on days 5–21 post laser treatment. Vascular leakage was measured via fluorescein angiography at ~9–10 mins after i.p. injection of fluorescein (late phase). Consistent with a previous report (37), laser photocoagulation induced CNV in WT C57BL/6 mice began to develop on day 3, reaching a maximum on day-5 post laser treatment. However, SD-OCT revealed no significant differences in lesion size between WT and FasL^ΔCS/ΔCS^ mice at all days examined (days 5, 8, 14, and 21 post laser treatment) (**Figures 3A, B**). Fluorescein angiography revealed similar results, with no significant differences in the area of vascular leakage between WT and FasL^ΔCS/ΔCS^ mice at all days examined (**Figures 3C, D**). These data demonstrate that expression of only mFasL did not prevent infiltration of choroidal vessels or decrease the area of vascular leakage. However, quite unexpectedly, the FasL^ΔCS/WT^ mice displayed a significant reduction in lesion size (**Figures 3A, B**) and vascular leakage area (**Figures 3C, D**) at day 5, 8, and 14 post laser treatment.

**Figure 3:**
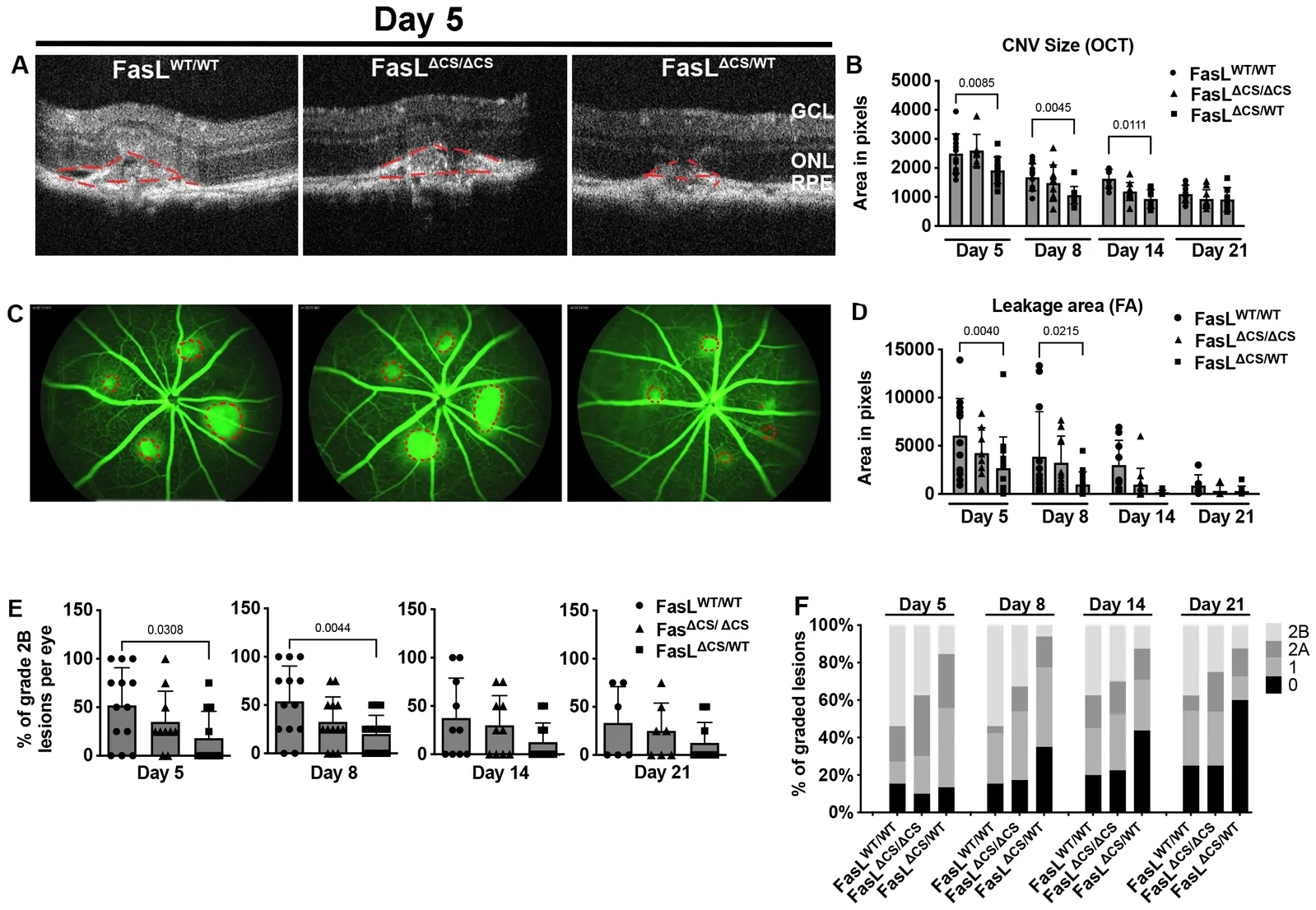
Expression of mFasL alone does not inhibit laser-induced CNV. **(A)** Representative OCT images acquired at day 5 post laser injury for FasL^WT/WT^, FasL ^ΔCS/ΔCS^, and FasL ^ΔCS/WT^ mice (Scale bar = 0.1mm). **(B)** Quantification of CNV size (red outline) by SD-OCT at Day 5, 8, 14, and 21 post laser injury. Shown is the mean area in pixels ± SD, N = 7–12 mice (D5), 10–12 mice (D8), 7–10 mice (D14), and 7–10 mice (D21), from 3 independent experiments. **(C)** Representative FA images acquired at day 5 post laser injury for FasL^WT/WT^, FasL^ΔCS/ΔCS^, and FasL ^ΔCS/WT^ mice. **(D)** Quantification of the area of leakage (red outline) by FA at 5, 8, 14, and 21 days post laser injury. Shown is the mean area in pixels ± SD, N = 8–15 mice (D5), 13–15 mice (D8), 9–12 mice (D14), and 6–8 mice (D21), from 3 independent experiments. **(E)** CNV lesions were analyzed for active leakage using a previously established grading scheme (38). The percent of 2B lesions (clinically significant leakage) was quantitated at Day 5, 8, 14, and 21 days post laser. Shown is the percent of grade 2B lesions per eye ± SD, N = 10-13mice (D5), 13–15 mice (D8), 10–12 mice (D14), 6–10 mice (D21), from 3 independent experiments. **(F)** The percent of all lesions within each grade (0,1, 2A, and 2B) were quantitated at 5, 8, 14, and 21 days post laser in FasL^WT/WT^, FasL^ΔCS/ΔCS^, and FasL^ΔCS/WT^ mice. P-values refer to 2-way ANOVA and Bonferroni’s multiple comparisons analysis.

An alternative method to quantify vascular leakage uses fluorescein angiography (FA) and evaluates the increase in area and intensity of fluorescence at three time points: initial phase at ~1–2 min, middle phase at 4–5 min and late phase at ~ 9–10 min post fluorescein injection. The advantage of this technique is that it reflects active leakage from the lesions and, therefore, is believed to be a more clinically relevant assessment of vascular leakage (38, 41). A previously established grading system was used to categorize the level of leakage as: none, minimal, moderate, and severe leakage (Grade 0, 1, 2A, and 2B, respectively) (38, 41). As seen in the previous analysis, there was no significant difference between WT and FasL^ΔCS/ΔCS^ mice at any time point for grade 0, 1, 2A, and 2B lesions (**Figures 3E, F**). However, at days 5, 8, and 14 post laser treatment FasL^ΔCS/WT^ mice displayed significantly fewer grade 2B lesions (severe leakage) as compared with WT and FasL^ΔCS/ΔCS^ mice (**Figures 3E, F**). Thus, we detected a significant reduction in vascular leakage in FasL^ΔCS/WT^ mice, but not FasL^ΔCS/ΔCS^ mice, using two different methods. We conclude that FasL is capable of inhibiting both the size of CNV, as measured via SD-OCT, and the amount of vascular leakage, as measured by fluorescein angiography. However, this inhibition did not occur in homozygous FasL^ΔCS/ΔCS^ mice, where FasL cleavage was completely prevented, but only in heterozygous FasL^ΔCS/WT^ mice.

### 3.2 Reduced vascular leakage in FasL^ΔCS/WT^ mice correlates with enhanced vascular maturity

We expected the reduced leakage observed in the FasL^ΔCS/WT^ would be due to FasL-mediated inhibition of vessels growing into the lesion. To examine this, choroidal flat mounts were prepared from FITC-dextran perfused mice at 3 and 5 days post laser injury and stained for collagen IV (**Figure 4**). Collagen IV, a vascular basement membrane marker, was used to identify the CNV lesion (42, 43) while FITC-dextran was used to label all perfused vessels. Contrary to our expectation, there was no significant difference in collagen IV accumulation between the three groups of mice (WT, FasL^ΔCS/ΔCS^ and FasL^ΔCS/WT^) at either 3 or 5 days post laser injury (**Figure 4A** middle row, **Figures 4B, C** middle row, and **Figure 4D**). Moreover, there was an increase in the presence of FITC-dextran perfused vessels growing into the lesions of FasL^ΔCS/WT^ mice. FITC-dextran perfused vessels began to appear in FasL^ΔCS/WT^ mice as early as 3 days post laser injury (**Figure 4A** top row and **Figure 4B**) and by 5 days post laser injury there was a significant increase in the area of FITC-dextran perfused vessels in FasL^ΔCS/WT^ mice compared to WT and FasL^ΔCS/ΔCS^ mice (**Figure 4C** top row and **Figure 4D**). Remarkably, perfused vessels in FasL^ΔCS/WT^ mice exhibited a more complex vascular network with increased branching as shown by a significant increase in vascular intersections as compared to the other groups (**Figure 4C** white arrows and **Figure 4E**). Together, these data support the conclusion that the decreased vascular leakage observed in CNV lesions of FasL^ΔCS/WT^ mice is due to enhanced vascular maturation and/or vessel normalization.

**Figure 4:**
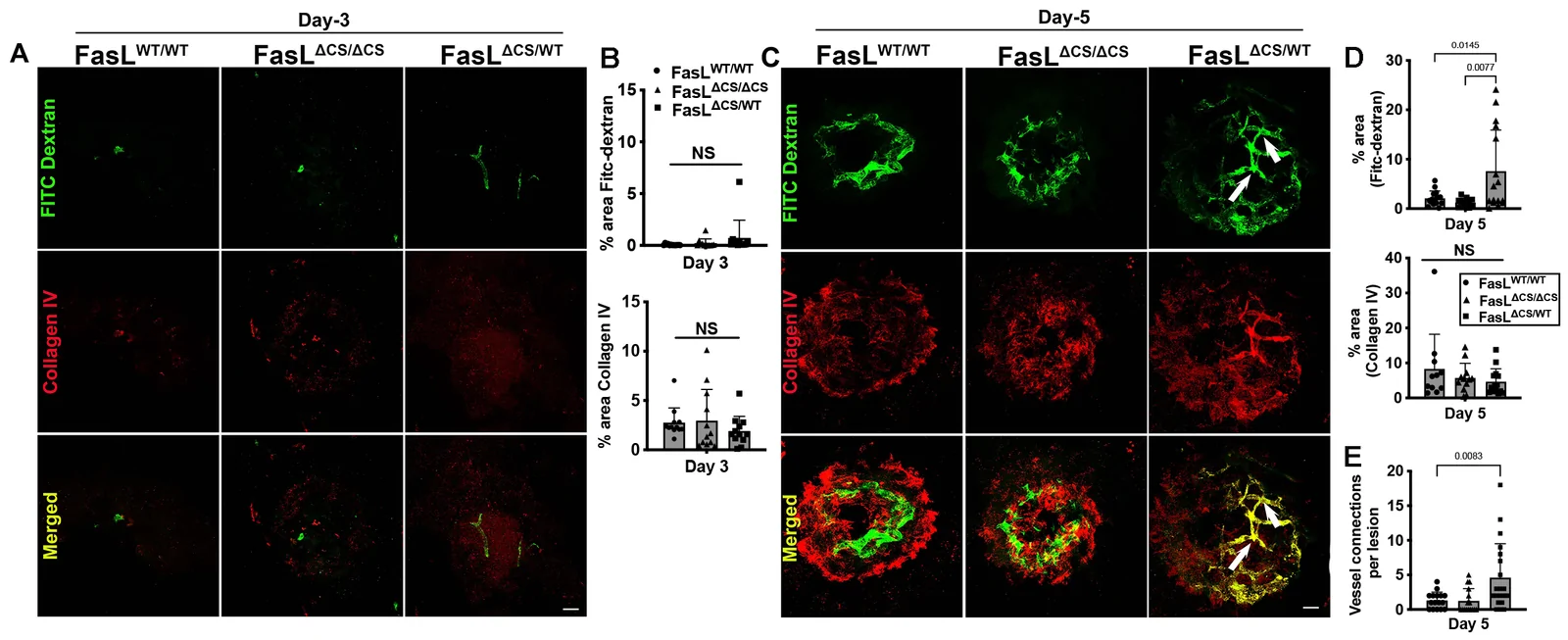
Analysis of neovessel structure. **(A, C)** Representative images from confocal analysis performed on FITC dextran (green) perfused RPE/Choroidal whole mounts stained for collagen IV (red) at Day 3 and Day 5 post laser (Scale bar =20μm) (40x magnification). **(B, D)** The area of FITC dextran staining (green) and area of collagen IV staining (red) was quantified in Z-stacked confocal images at **(B)** 3 days post laser and **(D)** 5 days post laser. Shown is the percent area ± SD, N = 12–16 CNV lesions from 3–4 mice per group. **(E)** The number of vascular intersections (white arrows) was quantified per lesion. Shown is the mean number of vessel connections per lesion ± SD, N = 12–16 lesions from 4 mice per group. P-values refer to 2-way ANOVA and Bonferroni’s multiple comparisons analysis.

Another hallmark of neovessel maturation is the formation of vascular endothelial cell tight junctions (44). These tight junctions can be most effectively visualized at the ultrastructural level by electron microscopy (EM). To further study neovascular formation/maturation of the CNV lesions, EM was used to assess endothelial tight junctions and lumen formation in the branching vessels of CNV lesions from 5 days post-laser. CNV lesions were located with a Toluidine blue stain of semi-thin sections (**Figure 5A**), and thin sections made from the lesion site were prepared for EM. Vascular endothelial cells were identified by the presence of their hallmark structures called Wiebel-Palade bodies (45). Outlines of vascular endothelial cells observed within CNV lesions indicated a disorganized endothelial cell pattern in FasL^WT/^WT (**Figure 5C**). By contrast, endothelial cells within CNV lesions from FasL^ΔCS/WT^ mice displayed a more organized branching morphology (**Figure 5B** bottom row, red dotted outline). In addition, the FasL^ΔCS/WT^ endothelial cells demonstrated tight junctions as shown by formation of kissing complexes, a hallmark of tight junctions, with high electron dense areas (46) (boxed areas in **Figure 5C** and red arrows in **Figure 5D**) that were absent from the endothelial cell junctional areas in CNV lesions from WT and FasL^ΔCS/ΔCS^ mice (red arrows, **Figure 5D**). In summary, CNV lesions of FasL^ΔCS/WT^ mice contained an increased number of perfused vessels, formation of complex vascular networks, and the presence of endothelial cell tight junctions. Together, these data support the conclusion that the decreased vascular leakage observed in CNV lesions of FasL^ΔCS/WT^ mice is due to enhanced vascular maturation and/or vessel normalization.

**Figure 5:**
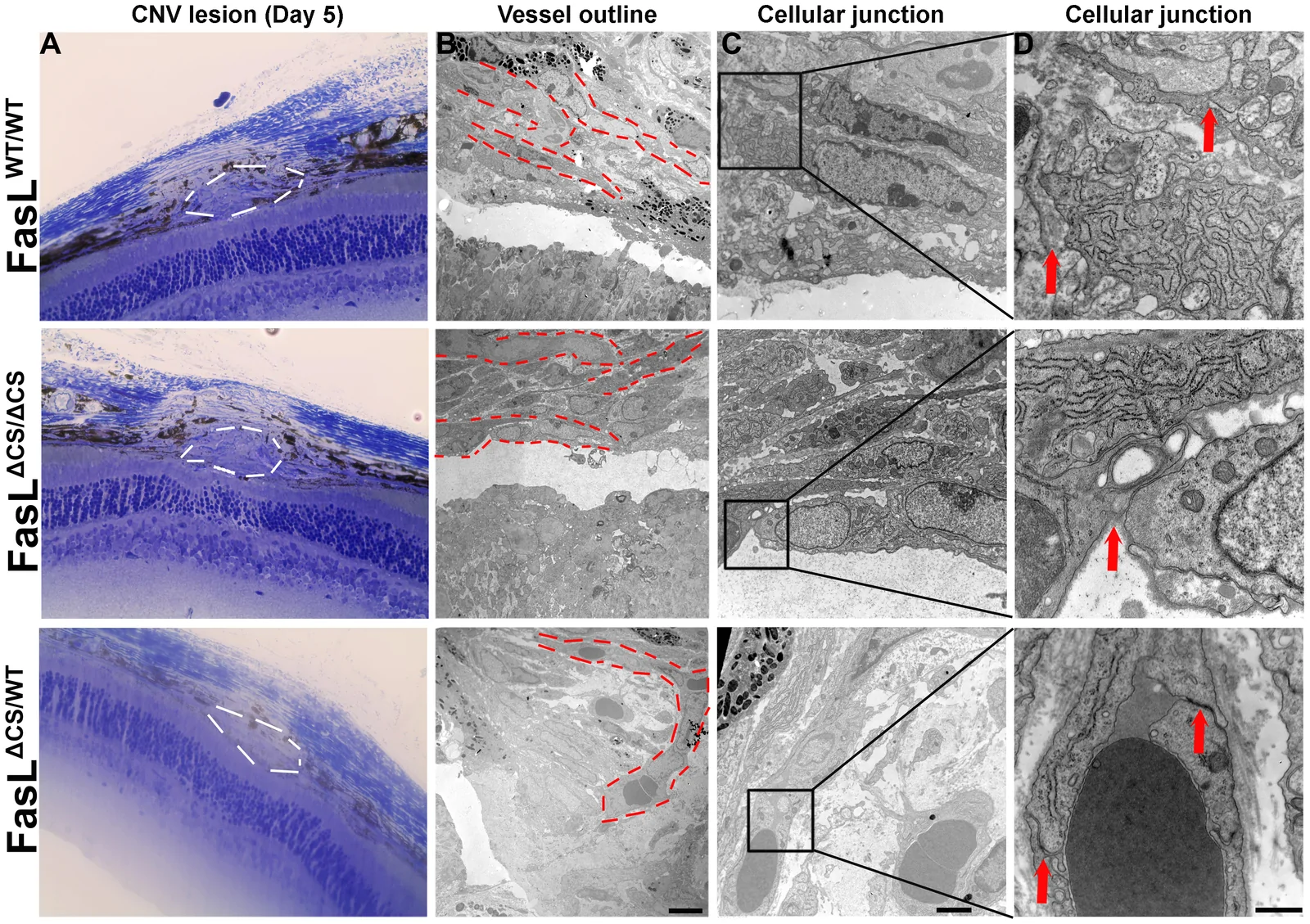
Analysis of vascular maturity by Electron microscopy (EM). **(A)** At 5 days post laser, Toluidine blue staining was performed to identify the start of the CNV lesion (white dotted line) prior to taking sections for EM analysis. **(B)** Representative EM images of the CNV lesion show developing vessels (red dotted outline) at 5 days post laser (1250x) and **(C)** cell-to-cell connections (in black box). **(D)** Higher magnification (red arrows) (49,000x) of the area in the black box shows the presence of tight junctions as demonstrated by kissing complexes with electron dense deposits in FasL ^ΔCS/WT^ mice, but not FasL^WT/WT^, or FasL ^ΔCS/ΔCS^ mice. Representative EM images from 3 mice per group. (Scale bars: panel B = 10μm, C = 2μm, D = 1μm).

### 3.3 PDGFRb, TGFβ1 and pericyte co-localization with perfused vessels

Pericytes, which envelop newly forming vascular tubes, promote vessel normalization by inhibiting endothelial cell proliferation, migration, and production of VEGF-R2 (47, 48). This process begins when sprouting endothelial cells release the chemotactic factor PDGFb (platelet derived growth factor beta) that in turn recruits PDGFRb (platelet derived growth factor Beta receptor) positive pericyte progenitor cells to the site of angiogenesis (49). Endothelial cells also release TGFβ1 that induces pericyte progenitors to differentiate into mature pericytes (48).

Therefore, one potential mechanism to accelerate vessel normalization and reduce vascular leakage would be to increase pericyte co-localization with perfused vessels via increased recruitment and maturation of pericyte progenitors. To examine pericyte co-localization with perfused vessels directly within developing CNV lesions, we stained FITC dextran-perfused choroidal flat mounts from FasL^WT/WT^, FasL^ΔCS/WT^, and FasL^ΔCS/ΔCS^ mice at 5 days post laser injury for NG2, a pericyte marker. Increased co-localization of NG2 positive cells (pericyte marker) with FITC dextran-perfused vessels was observed in regions showing increased branching (**Figures 6A–C**). While NG2 co-localization was not quantitated, we did quantitate the increased presence of more complex/mature vascular networks within the CNV lesions of FasL^ΔCS/WT^ mice as demonstrated by a significant increase in vascular intersections as compared to the other groups (**Figures 4C, E**). Moreover, quantitative real time PCR on posterior eyecups (retina, RPE, choroid and sclera) isolated from FasL^WT/WT^, FasL^ΔCS/WT^, and FasL^ΔCS/ΔCS^ mice at 5 days post laser injury revealed a significant increase in PDGFRb and TGFβ1 mRNA in FasL^ΔCS/WT^ versus WT mice, suggesting that FasL^ΔCS/WT^ mice can accelerate pericyte maturation in CNV lesions (**Figures 6E, F**). By contrast, no significant differences in the levels of PDGFRb and TGFβ1 were observed between FasL^ΔCS/ΔCS^ and FasL^WT/WT^ mice (**Figures 6D–F**) and no significant difference in PDGFb levels was observed between FasL^WT/WT^, FasL^ΔCS/WT^, and FasL^ΔCS/ΔCS^ mice (**Figure 6D**). Together, these data further support the conclusion that the decreased vascular leakage observed in CNV lesions of FasL^ΔCS/WT^ mice is due to enhanced vascular maturation and/or vessel normalization.

**Figure 6:**
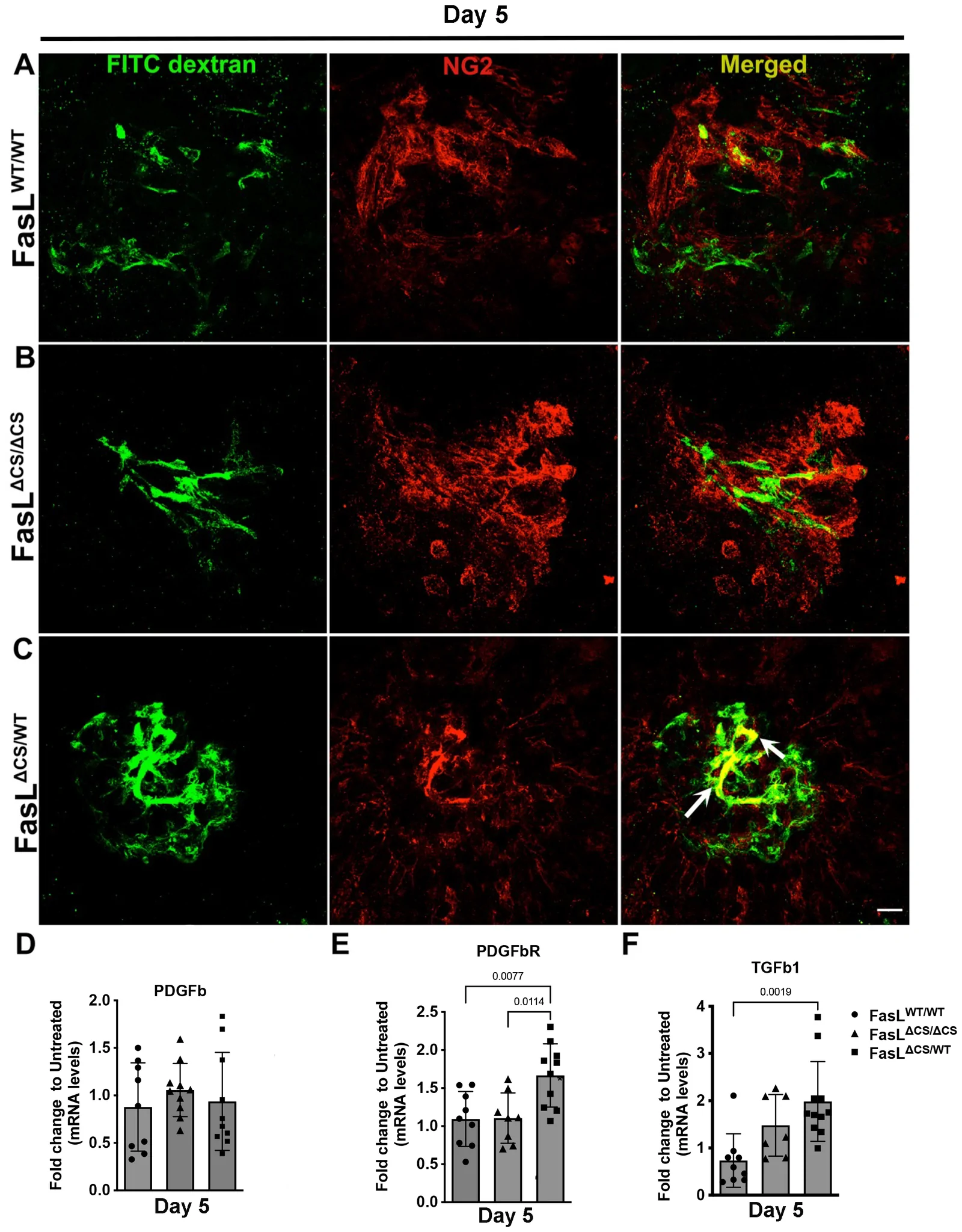
Co-localization of pericytes with perfused vessels and expression of PDGFb, PDGFRb, and TGFβ1. **(A–C)** Representative images from confocal analysis performed at 5 days post laser on FITC dextran (green) perfused RPE/Choroidal whole mounts stained for NG2 (red, pericytes marker) from FasL^WT/WT^ mice, FasL ^ΔCS/ΔCS^ mice, and FasL ^ΔCS/WT^ mice. Merged images show co-localization of NG2 with FITC dextran perfused vessels (yellow, white arrows) in FasL ^ΔCS/WT^ but not FasL^WT/WT^ or FasL ^ΔCS/ΔCS^ mice. (Scale bar-20μm) (40x magnification). Representative images from each group. **(D–F)** Quantitative PCR was performed on posterior eyecups at day-5 post laser to quantitate mRNA levels of PDGFb, PDGFbR, and TGF-β1. Data are shown as fold change from age-matched untreated controls ± SD. N = 7–11 mice per group from 3 independent experiments. P-values refer to one way ANOVA and Tukey’s multiple comparisons analysis.

### 3.4 VEGF, PEDF and vascular normalization

Vascular leakage is regulated, in part, by the ratio of vascular endothelial growth factor (VEGF) and pigment-epithelium derived factor (PEDF) in the retina (50–52). VEGFA in particular is one of the key factors controlling endothelial cell function in the eye (53) and the importance of VEGFA in vascular leakage is underscored by the clinical success of VEGFA neutralizing antibodies (bevacizumab, ranibizumab) in the treatment of wet AMD (10, 54–56). PEDF is a potent anti-angiogenic factor that has been shown to inhibit VEGF-induced vascular permeability both in vitro and in vivo (51, 52). To determine whether changes in the levels of VEGFA/PEDF coincided with the reduced vascular leakage in FasL^ΔCS/WT^ mice, we examined protein levels for VEGFA and PEDF using eyecups (retina, RPE, choroid) each containing 4 CNV lesions obtained from WT, FasL^ΔCS/WT^, and FasL^ΔCS/ΔCS^ mice at 5 days post laser treatment (**Figures 7A, B**). Eyecups obtained from untreated mice (no laser treatment) were used as negative controls. The protein level of VEGF was significantly elevated in laser treated WT mice, but not in laser treated FasL^ΔCS/WT^ or FasL^ΔCS/ΔCS^ mice (**Figure 7A**). By contrast, PEDF protein levels were significantly increased in laser treated FasL^ΔCS/WT^ mice but not WT or FasL^ΔCS/ΔCS^ mice (**Figure 7B**). No significant differences in VEGFA and PEDF protein levels were observed between WT and FasL^ΔCS/ΔCS^ mice (**Figures 7A, B**). Interestingly, VEGFA is not significantly elevated in FasL^ΔCS/WT^ or FasL^ΔCS/ΔCS^ mice, indicating that changes in VEGFA-alone cannot explain the phenotype we observe in FasL^ΔCS/WT^ mice. These data indicate that reduced vascular leakage within CNV lesions of FasL^ΔCS/WT^ mice coincides with both decreased levels of VEGFA and increased expression of PEDF, which is known to inhibit VEGF-induced vascular permeability and thereby promote vessel normalization.

**Figure 7:**
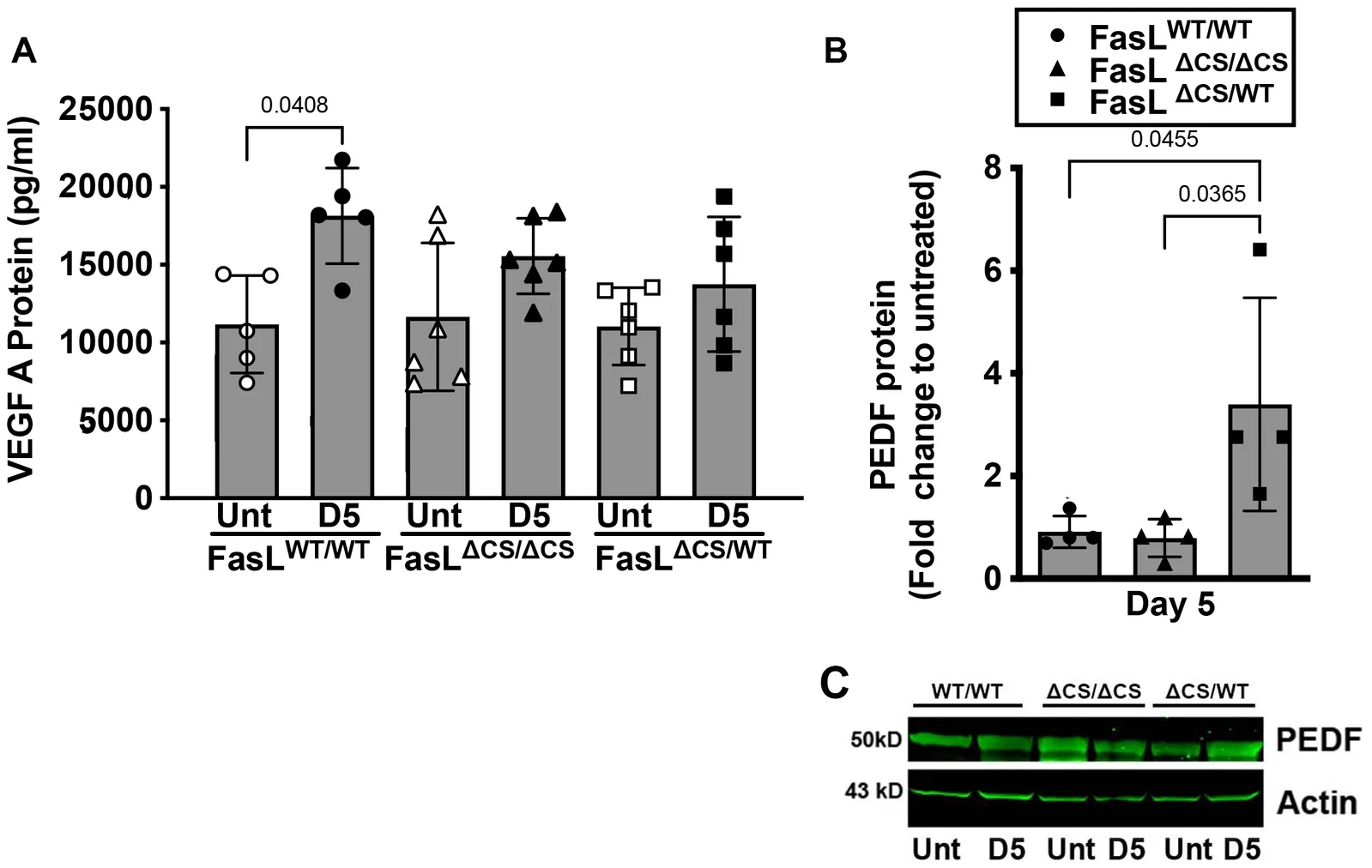
Proangiogenic and antiangiogenic protein expression analysis. **(A)** ELISA was performed to quantitate protein levels of VEGFA in posterior eyecups taken at day 5 post laser. Data are shown as mean pg/ml of VEGFA protein ± SD per posterior eyecup, N = 5–6 mice per group from 2 independent experiments. **(B)** Western blot was performed to quantitate protein levels of PEDF. **(C)** Representative Western blot of protein lysates prepared from posterior eye cups. Data are shown as fold change from untreated (Day 0) ± SD. N = 4 mice per group from 2 independent experiments. The uncropped Western blot is also provided (**Supplementary Figure 1**). Unt = untreated (no laser) controls which were genotype-, age-, and gender-matched to the laser-treated animals. P-values refer to one way ANOVA and Tukey’s multiple comparisons analysis.

### 3.5 Fas-FasL signaling in myeloid cells dictate neovascularization

Vascular normalization is an emerging strategy to enhance cancer immunotherapy, and several cancer studies indicate that vascular normalization is mediated in part by tumor-associated macrophages (57–61). Moreover, work by Apte et al., demonstrate that macrophages are protective against the development of CNV in the eye (62). Therefore, we asked whether Fas expressing myeloid cells mediate the protective phenotype observed in heterozygous FasL^ΔCS/WT^ mice. To address this question, heterozygous FasL^ΔCS/WT^ mice that conditionally lack Fas only in the myeloid cell compartment (FasL^ΔCS/WT^ Fas^flox/flox^ Cre^LysM^) were generated. PCR confirmed deletion of the Fas gene specifically within CD11b^+^-sorted splenic myeloid cells from FasL^ΔCS/WT^ Fas^flox/flox^ Cre^LysM^ mice (**Figure 8A**). To determine whether Fas expressing myeloid cells mediated vascular normalization in heterozygous FasL^ΔCS/WT^ mice, we induced CNV in FasL^ΔCS/WT^ Fas^flox/flox^ Cre^LysM^ mice that lack Fas in the myeloid compartment and compared to FasL^WT/WT^ and FasL^ΔCS/WT^ mice that do express Fas in the myeloid compartment. As a control for the effect of Fas^flox/flox^, we used FasL^ΔCS/WT^ Fas^flox/flox^ mice that did not express LysMCre. Following laser injury, we determined the size of CNV by SD-OCT and the area of vascular leakage by fluorescein angiography on days 5–21 post laser treatment. Consistent with **Figure 3**, the area of leakage and lesion size was significantly reduced in FasL^ΔCS/WT^ mice when compared FasL^WT/WT^ mice (**Figures 8B–D**). In the absence of Cre, the FasL^ΔCS/WT^ Fas^flox/flox^ mice showed no significant difference in the area of leakage and lesion size when compared to FasL^ΔCS/WT^ mice. However, in absence of Fas expressing myeloid cells, FasL^ΔCS/WT^ Fas^flox/flox^ Cre^Lysm^ mice presented with significant vascular leakage and CNV lesions equal to those observed in WT controls. Taken together, these data suggest Fas expressing myeloid cells mediate the vascular normalization observed in heterozygous FasL^ΔCS/WT^ mice.

**Figure 8:**
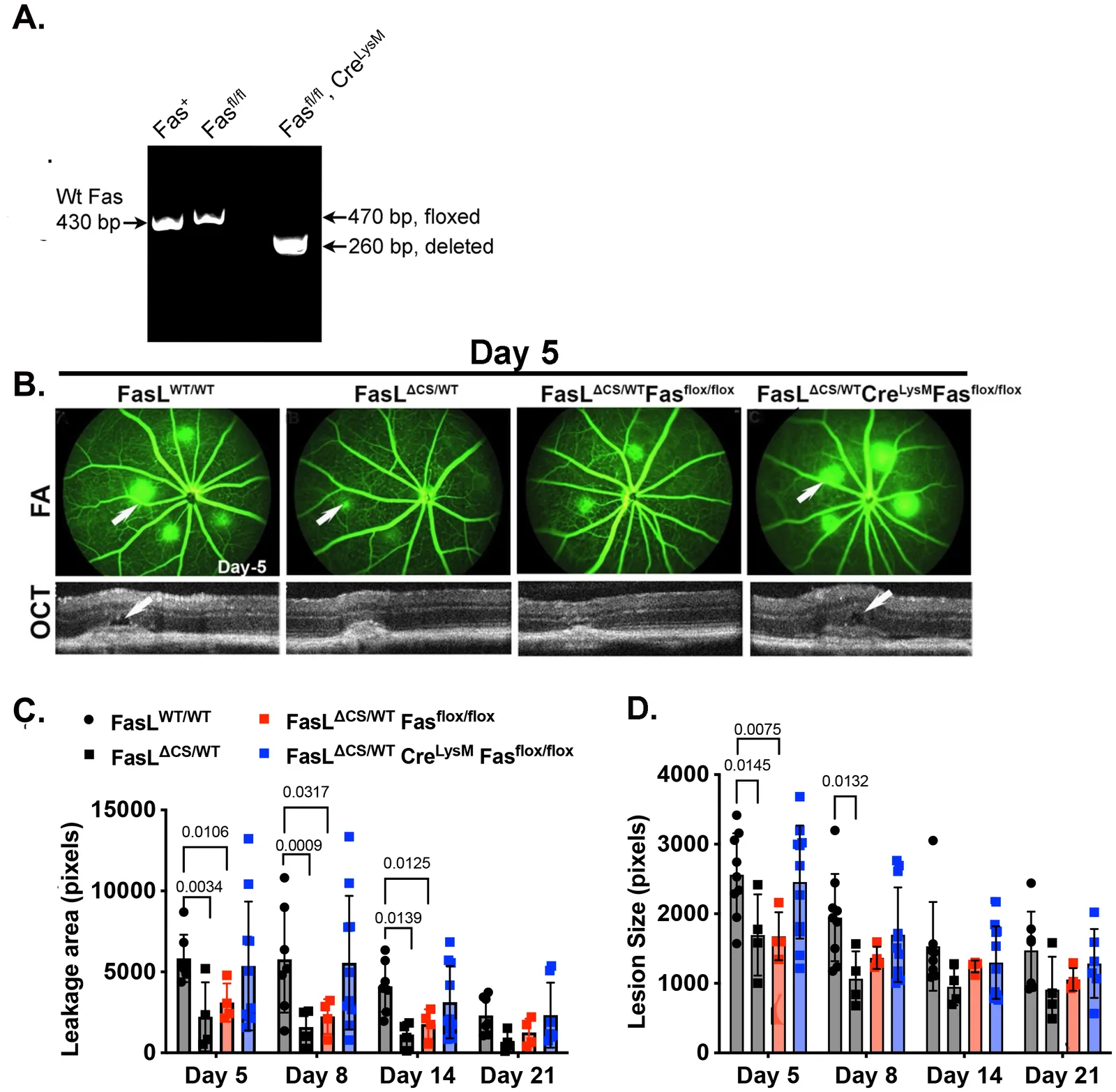
Fas expression on myeloid cells mediates protective phenotype in FasL ^ΔCS/WT^ mice. **(A)** Validation of FasL^ΔCS/WT^ Fas^flox/flox^ Cre^LysM^ mice. RT-PCR was performed on CD11b^+^-sorted splenic myeloid cells from FasL^ΔCS/WT^ mice expressing WT Fas (FasL^ΔCS/WT^, Fas^+^), floxed Fas (FasL^ΔCS/WT^, Fas^fl/fl^), or floxed Fas and LysMCre (FasL^ΔCS/WT^,Fas^fl/fl^, Cre^LysM^). Bands: Fas^Wt-allele^ (430bp), Fas^floxed-allele^ (470bp), and Fas^deleted-allele^ (260bp). **(B)** Representative FA and OCT images acquired at day 5 post laser injury for FasL^WT/WT^, FasL^ΔCS/WT^, FasL^ΔCS/WT^.Fas^fl/fl^, and FasL^ΔCS/WT^.CreLysM, Fas^fl/fl^ mice (white arrows show area of leakage in FA and OCT). **(C)** Quantification of the area of leakage by FA at 5, 8, 14, and 21 days post laser injury. Shown is the mean area in pixels ± SD. FasL^WT/WT^ (N = 7), FasL^ΔCS/WT^ (N = 4), FasL^ΔCS/WT^,Fas^fl/fl^(N = 4), and FasL^ΔCS/WT^,CreLysM, Fas^fl/fl^ (N = 7-10). **(D)** Quantification of CNV size by SD-OCT at Day 5, 8, 14, and 21 post laser injury. Shown is the mean area in pixels ± SD. FasL^WT/WT^ (N = 7-9), FasL^ΔCS/WT^ (N = 4), FasL^ΔCS/WT^,Fas^fl/fl^(N = 4), and FasL^ΔCS/WT^.CreLysM, Fas^fl/fl^ (N = 6-11). P-values refer to 2-way ANOVA and Bonferroni’s multiple comparisons analysis.

## 4 Discussion

Choroidal neovascularization is the hallmark of wet AMD where abnormal choroidal vessels infiltrate into the outer retina of the macula. These new blood vessels often bleed and leak fluid, causing retinal swelling and detachment with subsequent loss of vision. Fas ligand (FasL) is constitutively expressed in the eye and has been implicated in the inhibition of neovascularization in several *in vivo* models of neovascularization (suture-induced, hyperoxia-induced, and laser-induced), where increased neovascularization was observed in FasL deficient mice (gld/gld) (20, 63, 64). In all cases, it was concluded that FasL controlled neovascularization by inducing apoptosis of infiltrating Fas+ endothelial cells. Moreover, in 2006, Semkova et al. used a transgenic model to demonstrate that specific overexpression of FasL on retinal pigment epithelial (RPE) cells prevented laser-induced CNV (22) thereby identifying the Fas-FasL pathway as a potential therapeutic target in the treatment of CNV.

Numerous studies from our lab and others have found that mFasL is pro-apoptotic and pro-inflammatory, while sFasL is non-apoptotic and anti-inflammatory (15, 28–30, 32, 65–70). It followed that the relative levels of the soluble and membrane forms of FasL in the eye at any given time reflect the extent of ongoing ocular pathologies. Notably, in a healthy eye, most FasL exists as a soluble protein, sFasL, while in a glaucomatous eye FasL is no longer cleaved and exists as membrane-bound protein, mFasL, that promotes the death of retinal ganglion cells. Moreover, FasL^ΔCS/ΔCS^ mice that only express mFasL exhibit much more severe retinal damage in both genetic and inducible murine models of glaucoma (30, 39). Therefore, if CNV is directly constrained by mFasL-mediated apoptosis of infiltrating choroidal endothelial cells (21–23), then laser-induced CNV in FasL^ΔCS/ΔCS^ mice should result in smaller CNV lesion size and less extensive vascular leakage. However, we unexpectedly found that there was no significant difference in either the lesion size or the area of leakage between FasL^WT/WT^ mice and homozygous FasL^ΔCS/ΔCS^ mice following laser treatment. Moreover, the CNV lesions in both FasL^WT/WT^ and FasL^ΔCS/ΔCS^ mice contained disorganized, immature, and leaky vessels, characterized by poor pericyte coverage and lack of proper tight junctions between endothelial cells, similar to the sprouting microvessels found in patients with neovascular AMD that are immature, dilated, and lack organization or hierarchy (71, 72). These data indicate that in the absence of FasL cleavage, mFasL alone is unable to prevent the development of laser induced CNV.

However, most importantly, our experiments revealed a very unexpected and unique vascular phenotype in heterozygous FasL^ΔCS/WT^ mice; these mice displayed a significant reduction in lesion size and area of vascular leakage, as compared to either FasL^WT/WT^ or FasL^ΔCS/ΔCS^ mice. Current dogma would predict that the smaller lesions in heterozygous FasL^ΔCS/WT^ mice would coincide with a reduced ingrowth of choroidal vessels into the retina. Yet, we observed the opposite outcome; CNV lesions in the heterozygous FasL^ΔCS/WT^ mice contained numerous choroidal vessels that were highly organized, well perfused, and displayed normal pericyte coverage and tight junctions between endothelial cells, both hallmarks of mature vessels (58, 73). These results suggest that the prior doxycycline studies (33) resulted from only partial inhibition of FasL cleavage. More importantly, the data clearly demonstrate that the extent of FasL cleavage has a dramatic impact on how FasL regulates CNV development. However, while a genetic approach was used to specifically control the extent of mFasL cleavage, future studies will assess the extent, timing, and tissue-specific expression of mFasL and sFasL in the three cohorts of mice (FasL^WT/WT^, FasL^ΔCS/WT^, FasL^ΔCS/ΔCS^) following laser-induced CNV to identify the source(s) of FasL and determine the optimal balance of mFasL and sFasL required for the protective phenotype.

In physiologic angiogenesis, newly formed vessels mature quickly, stabilize, and stop proliferating. However, in pathologic angiogenesis, such as in neovascular AMD, an imbalance of anti- and pro-angiogenic factors results in the uncontrolled growth of neovessels that are abnormal and leaky, and under constant reconstruction (74). The high VEGF (pro-angiogenic) to PEDF (anti-angiogenic) ratio expressed in the eyes of WT mice following laser injury, correlated with CNV lesions characterized by leaky immature vessels that lack proper pericyte coverage, resulting in significant vascular leakage. By contrast, the significant increase in PEDF expression detected in the heterozygous FasL^ΔCS/WT^ mice correlated with the formation of mature, non-leaky vessels.

Studies of tumor vascular development demonstrate that it is possible to prevent pathologic angiogenesis by restoring the angiogenic balance thereby promoting vessel maturation and normalization (57, 60, 61). The “Vascular Normalization Hypothesis” states that direct or indirect anti-angiogenic therapy, aimed at inhibiting pro-angiogenic factors and upregulating anti-angiogenic factors restores the angiogenic balance (57, 61). As a result, vessels mature with increased pericyte coverage, proper endothelial cell tight junctions, normal perfusion, and reduced vascular leakage (48, 57).

Our current data points to a critical balance between too much or too little FasL cleavage and we propose this may be a “Goldilocks-like” effect that reflects subtle but important differences in FasL function that depends on the level of mFasL expression. For example, too much mFasL in the FasL^ΔCS/ΔCS^ mice may favor the pro-apoptotic activity of mFasL and lead to the death of critical PDGFRb, TGFβ1 or PEDF -producing cells, while the optimal expression of mFasL in FasL^ΔCS/WT^ mice may favor cytokine production. Alternatively, both mFasL and sFasL may independently contribute to the regulation of neovascularization. Prior studies by Legembre and colleagues have shown that sFasL can promote cell migration (75) and may therefore contribute to the recruitment of pericyte precursors in AMD, while mFasL promotes cytokine production (76) in macrophages or other cell types and may promote the production of critical factors required for neovessel maturation. These questions will need to be addressed in future studies.

For now, we have shown that deletion of the Fas receptor on myeloid cells using an intercross of LysM-Cre and floxed-Fas strains eliminates the protective activity of FasL in the FasL^ΔCS/WT^ mice. These data point to the myeloid subset as the critical Fas target and potential source of factor(s) that stimulate vascular normalization in the FasL^ΔCS/WT^ mice. We and others have previously shown that FasL can induce the production of a variety of cytokines in Fas^+^ macrophages that are relatively insensitive to FasL-mediated apoptosis (76–78). Moreover, FasR engagement has been shown to drive M1-polarization in macrophages (79) and M1-macrophages are associated with vessel normalization (59, 80, 81). However, it is not immediately clear why FasL^ΔCS/ΔCS^ myeloid cells would not serve a similar function. We propose this dichotomy may reflect a “Goldilocks-like” effect we discussed above – the stronger FasL signal in FasL^ΔCS/ΔCS^ mice may lead to the demise of the Fas^+^ myeloid cells that in FasL^ΔCS/WT^ mice produce the factors that promote neovessel maturation.

One limitation to this study is that LysM-Cre targets multiple myeloid populations, including macrophages, monocytes, and neutrophils, so while our data establishes a connection between Fas signaling within the LysM-expressing myeloid compartment and the protective phenotype observed in the FasL^ΔCS/WT^ mice, we cannot distinguish among macrophages, monocytes, and neutrophils. Future studies using more-cell-specific approaches will be required to identify the specific CNV-associated myeloid population(s) that mediate the protective phenotype. In addition, in this study we used young adult (10–12 weeks) mice and while young adult mice are commonly used in laser-induced CNV studies, AMD is an aging-associated disease and age-related changes in the choroidal immune environment, macrophage phenotype and function, RPE responses, and vascular repair, may also influence FasL-mediated responses. Moreover, Kelly et al. demonstrated that eyes of aged mice were enriched for pro-angiogenic, M2 macrophages (82). Therefore, while our findings demonstrate a protective phenotype in young FasL^ΔCS/WT^ heterozygous mice, future studies in aged mice will be necessary to determine if this protective phenotype persists with age.

## 5 Conclusion

In conclusion, our data are not consistent with a purely mFasL-mediated endothelial cell apoptosis model in CNV. Rather our data suggest that FasL may mediate inhibition of vascular leakage in the mouse model of AMD through *non*apoptotic pathways that promotes vascular maturation mediated by LysM-expressing myeloid cells and this effect is highly dependent on the extent of FasL cleavage as only heterozygous FasL^ΔCS/WT^ mice, and not homozygous FasL^ΔCS/ΔCS^ mice, exhibited reduced CNV. Future studies will use more-cell-specific approaches to precisely identify the CNV-associated myeloid population(s) that mediate the protective phenotype and determine whether sFasL and mFasL can drive macrophage polarization and regulate neovessel maturation.

However, an important point to keep in mind is that wet AMD consists of two distinct phases, (i) choroidal neovascularization and (2) subretinal fibrosis (40, 83) and it has been reported that a significant number of patients receiving long-term anti-VEGF therapy develop significant vison loss within 5 years due to atrophy and the development of subretinal fibrosis (84, 85). Therefore, while accelerated maturation/normalization of neovessels may reduce vascular leakage and prevent vision loss in the early stages of wet AMD, it will also be important to determine if persistent “non-leaky” CNV contributes to increased subretinal fibrosis. In addition, a recent study demonstrates a sex difference in the inflammatory response to laser-induced CNV, with male mice displaying a more inflammatory phenotype as compared to female mice (86). While males and females were balanced in each experiment herein, our study overall was not sufficiently powered to assess specific sex differences, and this should be considered in future studies.

## Data Availability

The raw data supporting the conclusions of this article will be made available by the authors, without undue reservation.
